# Characterization of the Oral Microbiome Among Children With Type 1 Diabetes Compared With Healthy Children

**DOI:** 10.3389/fmicb.2021.756808

**Published:** 2021-10-29

**Authors:** Moti Moskovitz, Mira Nassar, Nadav Moriel, Avital Cher, Sarit Faibis, Diana Ram, David Zangen, Moran Yassour, Doron Steinberg

**Affiliations:** ^1^Department of Pediatric Dentistry, Faculty of Dental Medicine, Hadassah Medical Center, The Hebrew University of Jerusalem, Jerusalem, Israel; ^2^Biofilm Research Laboratory, Faculty of Dental Medicine, Institute of Dental Sciences, The Hebrew University of Jerusalem, Jerusalem, Israel; ^3^Microbiology and Molecular Genetics Department, Faculty of Medicine, The Hebrew University of Jerusalem, Jerusalem, Israel; ^4^Division of Pediatric Endocrinology, Hadassah Medical Center, The Hebrew University of Jerusalem, Jerusalem, Israel

**Keywords:** type 1 diabetes, children, 16S rRNA gene sequencing, salivary microbiome, periodontitis

## Abstract

**Aim:** Current microbiome profiling of type 1 diabetes mellitus (T1D) patients is mostly limited to gut microbiome. We characterized the oral microbiome associated with T1D in children after the onset of the disease and explored its relationship with oral physiological factors and dental status.

**Methods:** This cohort study comprised 37 children aged 5–15 years with T1D and 29 healthy children matched in age and gender. Unstimulated whole saliva was collected from diabetic and non-diabetic children, in the morning after brushing their teeth and a fasting period of at least 1 h before sampling. 16S rRNA gene-based analysis was performed by Powersoil Pro kit by Qiagen and Phusion High-Fidelity PCR Master Mix. Oral physiological and dental parameters studied included decayed, missing, and filled teeth index, salivary flow rate, and salivary pH, glucose, calcium, phosphate, and urea levels.

**Results:** Of the identified 105 different genera and 211 different species, the most abundant genera were *Streptococcus*, *Prevotella*, *Veillonella*, *Haemophilus*, and *Neisseria*. *Streptococcus* was more abundant in T1D children. The diabetes group had 22 taxa at the genus level and 33 taxa at the species level that were not present in the control group and the control group exhibited 6 taxa at the genus level and 9 taxa at the species level that did not exist in the diabetes group. In addition, *Catonella*, *Fusobacterium*, and *Mogibacterium* differed between healthy and T1D subjects. Eight species and eight subspecies were significantly more abundant among healthy children than in T1D children. *Porphyromonas* and *Mogibacterium* genera were significantly correlated with salivary parameters. We found similarities between taxa revealed in the present study and those found in gut microbiome in type 1 diabetes mellitus according to gutMDisorder database.

**Conclusions:** Salivary microbiome analysis revealed unique microbial taxa that differed between T1D children and healthy subjects. Several genera found in the saliva of T1D children were associated with gut microbiome in T1D individuals.

## Introduction

Oral microbiome represents an important part of the human microbiome and can have detrimental consequences on both our general and oral health. The genetic setup of the host may affect the microbial composition and function, the activation of intrinsic and adaptive immunity, and susceptibility to various diseases (Zhou et al., 2020). Accumulating evidence links oral bacteria to several systemic diseases including diabetes (Genco et al., 2005). Type 1 diabetes (T1D), also known as insulin-dependent diabetes, is a chronic autoimmune-mediated disease in which the insulin-producing pancreatic beta cells are destroyed. Although it can be diagnosed at any age, it is one of the most common chronic diseases of childhood and adolescence (Maahs et al., 2010; Hong et al., 2017). T1D accounts for 5–10% of diabetic patients worldwide (Maahs et al., 2010; World Health Organization, 2018) and is the second most frequent autoimmune disease in childhood; its incidence has tripled in the last 30 years (de Groot et al., 2017). Worldwide, 1.1 million children and adolescents under the age of 20 live with T1D (International Diabetes Federation, 2019).

The increasing disease rate cannot be explained merely by genetic factors but implies that these changes are an outcome of interactions between the environment and predisposing genes (Siljander et al., 2019).

Diabetes is associated with several soft-tissue abnormalities in the oral cavity secondary to the disease that have a significant effect on the quality of life of diabetic patients (Ferizi et al., 2018). Patients with T1D are more susceptible to periodontal diseases and tooth loss and such problems might be aggravated with aging (Sadeghi et al., 2017). Quantitative and qualitative salivary changes in diabetics have also been confirmed (Angus and Richard, 2008).

The oral microbiome is known to vary in response to oral and systemic diseases (Simpson and Thomas, 2016). Diabetes has a significant impact on the gut microbial composition, stability, and connectivity, which in turn can alter the development of T1D by influencing the immune response of hosts (Han et al., 2018). Oral microbiome of adults has been implicated in the development of type 2 diabetes (T2D), but has been rarely explored in T1D. Long et al. (2017) analyzed the oral microbiome of T2D patients and discovered that the relative abundance of *Actinobacteria*, which associates with a lower risk of developing T2D, decreased (Long et al., 2017). On the contrary, a study in T1D subjects showed significantly higher abundance of taxa belonging to the phyla *Actinobacteria* and *Firmicutes*, including *Streptococcus* spp., *Actinomyces* spp., and *Rothia* spp. (de Groot et al., 2017).

The complex etiology of T1D is underlined by the fact that several years may pass between initial β-cell damage to manifestation of clinical diabetes (Størling and Pociot, 2017). Thus, early diagnosis of diabetes by targeting the microbiota at the latent period could potentially enable early treatment and postpone T1D development in children with β-cell autoimmunity.

The aim of the present study was to profile the salivary microbiome of children with T1D based on 16S ribosomal RNA (16S rRNA) gene community profiling, and to compare it with healthy children, while considering additional aspects of the oral environment. We also analyzed the impact of oral and salivary parameters including DMFT index, salivary flow rate, glucose, pH, calcium, phosphate, and urea on the salivary microbiome.

## Materials and Methods

### Study Population

#### Ethical Considerations

All procedures performed were in accordance with the study protocol [ClinicalTrials.gov (NCT03908021)] that was approved by the Institutional Human Subjects Ethics Committee of Hadassah Medical Organization (0714-18-HMO). No compensation was provided for the participating patients. The study was conducted in the period from 2019 to 2020.

Because this was an initial study examining the differences in oral microbiome between children with T1D and non-diabetic children, no power calculation was performed. It was decided to collect saliva from all attendants to the division of Pediatric Endocrinology, Hadassah Medical Center, Hebrew University of Jerusalem, Israel, who met the inclusion criteria and were willing to participate in the study during a period of 1 year. Those children were matched in age and gender with healthy children attending the postgraduate program in Orthodontics of the Hebrew University–Hadassah Faculty of Dental Medicine. Control group saliva collection was terminated after a year.

The study was conducted on 66 children, including 37 with diabetes aged 5–15 years, during a routine follow-up visit at the Pediatric Endocrinology Clinic, Hadassah Hebrew University Medical Center (Jerusalem, Israel). All diabetic children were treated with but not with any other therapy at least a week prior to checkup. The control group, matched in age and gender, included 29 healthy children who were attending the Orthodontic Clinic at the same medical center. All healthy children were without functional orthodontic appliances and no history of drug therapy at least a week prior to checkup. Exclusion criteria for both groups were diseases other than T1D and known oral disease. All patients were medication free apart from insulin if needed at the day of sample collection and at least a week before.

### Clinical Examination and Collection of Saliva Samples

Clinical dental health status was measured using the Decayed, Missing and Filled Teeth (DMFT) Index according to the WHO caries diagnostic criteria for epidemiological studies (World Health Organization, 1997). All dental examinations were performed by a single qualified dentist from the department of Pediatric Dentistry, Faculty of Dental Medicine, Hebrew University of Jerusalem, Israel, in accordance with the clinic checkup procedures.

Access to dental care, parents’ dental education, and the quality of diet were provided through patients’ and parents’ interview.

Unstimulated whole saliva was collected from diabetic and non-diabetic children, in the morning after brushing their teeth and a fasting period of at least 1 h before sampling. The children were asked to spit saliva into a 15-ml sterile tube over a measured period of time and sufficient for salivary parameter measurement. Only the liquid of the saliva was allocated and collected for the analysis (He et al., 2015).

Before centrifugation, 350 μl of saliva was stored at −80°C for microbiome analysis, and pH and salivary glucose were determined. The saliva samples were then centrifuged at 1,500 RCF (relative centrifugal force) for 15 min at 4°C to reduce salivary debris and viscosity. Salivary calcium, phosphate, and urea were evaluated later in the supernatant fluid, stored at −20°C.

### Measurement of Salivary Flow Rate, pH, and Glucose

The salivary flow rate was defined without the foam as the volume (in ml) of saliva secreted per minute of collection. Salivary pH was measured using color-coded pH-indicator strips (pH 0–14 Universal indicator, MQuant; Sigma-Aldrich, Israel). Glucose test strips (Medi-Test Combi 3A; Praxisdienst, Germany) measured salivary glucose.

### Measurement of Salivary Calcium, Phosphate, and Urea

Salivary calcium, phosphate, and urea concentrations were calorimetrically measured from the stored clear salivary supernatant fluid and according to the manufacturer’s instructions. The following kits were used, respectively: Calcium Colorimetric Assay Kit (MAK022—Sigma-Aldrich, St. Louis, MO 63103, United States), Phosphate Colorimetric Assay Kit (MAK030—Sigma-Aldrich), and Amplite Colorimetric Urea Assay Kit ^∗^Blue Color^∗^ (10058—AAT Bioquest, Sunnyvale, CA 94085, United States).

### Microbiome Analysis and 16S Ribosomal RNA Gene-Based Analysis

DNA extraction was performed by the Powersoil Pro kit by Qiagen (47016), following the company’s protocol, with mild modifications. All saliva samples were centrifuged at 14,000 × *g* for 10 min at 4°C. The supernatant fluid was discarded and the pellet was re-suspended in 800 μl of CD1 and then added to the PowerBead Pro tube. Samples were also treated in a bead beaten beater (TissueLyzer; QIAGEN) at 20 Hz for 10 min. 16S rRNA libraries were prepared according to the published protocol (Poyet et al., 2019) with mild modifications. First, qPCR was used to normalize template concentrations and determine the optimal cycle number needed for amplification of the V4 region of the 16S rRNA gene. In the qPCR, each sample was amplified in two 25-μl reactions using iTaq Universal SYBR Green Supermix (#17525124) and the primers 515 F (AATGATACGGCGA CCACCGAGATCTACACTATGGTAATTGT GTGCCAGCMG CCGCGGTAA) and 806rcbc0 (CAAGCAGAAGACGGCATAC GAGAT TCCCTTGTCTCC AGTCAGTCAG CC GGACTACH VGGGTWTCTAAT). Samples were quantified using the formula 1.75^ΔCt^. To minimize over-amplification, each sample was diluted to the lowest concentration sample, and the Ct value of this lowest concentration sample was used as the cycle number in the PCR reaction for library construction.

For library construction, four 25-μl reactions were prepared per sample using Phusion High-Fidelity PCR Master Mix with HF buffer (M0531L) and the primer 515F and 806R. Each sample was given a unique reverse barcode primer from the Golay primer set (see “Ultra-high-throughput microbial community analysis on the Illumina HiSeq and MiSeq platforms”; Caporaso et al., 2012). The replicates were then pooled and cleaned using Agencourt AMPure XP beads. Purified libraries were diluted 1:100 and quantified via qPCR, again using two reactions of 25 μl with iTaq Universal SYBR Green Supermix, but with the primers Read 1 and Read 2. The undiluted samples were normalized by way of pooling using the formula mentioned previously, and the pools were quantified by Qubit, as well as analyzed on the TapeStation. The pools were then normalized into a final pool based on the concentration calculated by Qubit, the average library size determined by TapeStation results, and the number of samples in the pool.

Final pools were sequenced on an Illumina MiSeq using the custom index 5′-ATTAGAWACCCBDGTAGTCCGGCTGA CTGACT-3′ and custom Read 1 and Read 2, mentioned previously, and using 30% PhiX.

### 16S Ribosomal RNA Analysis

All sequences passed fastQC using default parameters and had an average of 11,500 reads (with a minimum of 5,876 reads per sequence). BURST v0.99 (Al-Ghalith and Knights, 2017) was applied to the raw reads using default parameters, and with the burst_linux_DB12 database,^1^ which is based on the RefSeq Targeted Loci Project.^2^

Results were then divided according to taxonomic level. For family, genus, species, and subspecies level, a threshold of reads was set to 25. Thus, only taxa which had more than 25 reads throughout all the samples were further analyzed. Following the removal of the bacteria that did not meet the threshold, the relative abundance of each bacterium in each sample was re-normalized. A taxon was considered abundant if it had a relative abundance greater than 5% in at least one sample.

### Statistically Significant Differential Taxonomic Analysis

To find differential bacterial taxa in which the relative abundance was statistically different between T1D patients and control subjects, we used MaAsLin 2 (Microbiome Multivariable Associations with Linear Models) (Mallick et al., 2021) multivariate linear regression along with an annotation of whether the sample belonged to a case or control. MaAslin2 results were considered statistically significant in case *q*-value < 0.25. Relevant taxa were then plotted using the ggplot2 package v3.3.3 (Wickham, 2016) in R v4.0.3. The plots include annotation of the coefficient, *p*-value, and *q*-value as calculated by MaAslin2.

#### Taxa Appearing Only in the Study or Control Group

To find bacteria at a certain taxonomic level that appeared only in one of the groups, we took the data table of the relevant taxonomic level and selected only the bacteria that were completely missing in one study group. The value represented by the axis refers to the sum of the relative abundances of the bacteria, within the samples of the axis’s population.

#### Microbial Taxa Association With Various Variables

To find significant associations between various subject parameters and specific bacteria, the data of the relevant taxonomic level was given as input to MaAslin2 alongside a table containing the various metadata variables. Results were considered significant if they had a *q*-value smaller than 0.25.

#### Heatmap of Abundant Taxa

A genus was considered abundant if it had a relative abundance greater than 5% in at least one sample across all analyzed samples. This added up to 23 abundant genera in our analysis. The relative abundance of each genus within each sample was plotted into a heatmap, along with an annotation at the top of the plot designating if the sample belonged to a case or control. The heatmap plot was created using the pheatmap package v1.0.12 (Raivo, 2019) in R v4.0.3.

### Alpha Diversity Analysis

Alpha diversity measurement was done using the Shannon diversity index that was calculated using the diversity function within the vegan v2.5.7 R package.^3^ A Wilcoxon test was applied between the two population groups using the ggpubr v0.4 package.^4^

### Principal Coordinate Analysis

Beta diversity was calculated using the Bray–Curtis dissimilarity index as calculated using the vegan 2.5.7 and ape 5.4.1 R packages (Paradis and Schliep, 2019).

### Data Analysis

The average and standard error (SE) of DMFT index, salivary flow rate, pH, glucose, calcium, phosphate, and urea between the two groups were analyzed using Student’s *t*-test and *p* < 0.05 was considered statistically significant.

Microbiome data were analyzed by the ‘‘Burst Analyzer’’ software (Burst-Analyzer—knights-lab)^5^ and MaAslin2 comprehensive R package (Maaslin2—Bioconductor)^6^ for efficiently determining specific genus and families in which considerable differences were found between study and control samples. Data visualization was performed by ggplot2 in R package (Wickham, 2016).

## Results

### Population

The study group included 37 children with T1D (17 males) with a mean age (±SD) of 13 ± 2.69 years, and the control group included 29 (11 males) healthy children with a mean age (±SD) of 10 ± 2.38 years with no other relevant differences noted between the groups. All study group participants were using insulin since diagnosed as having T1D; 81.1% of them used insulin pumps with continuous delivery of short-acting insulin. The mean (±SD) time since diagnosis of diabetes was 2 ± 2.58 years. According to the patients’ files, 70.3% of diabetic children were metabolically stable at the time of sample collection.

As looking into caries risk factors is beyond the scope of this preliminary study, we used only a general interview that is accepted for initial checkup in the department of Pediatric Dentistry, Faculty of Dental Medicine, Hebrew University of Jerusalem, Israel. A more comprehensive study that will address this issue is planned as a future project. Interviews revealed that children with T1D visited the dentist only when necessary, while children in the control group were orthodontic patients who kept high standards of oral care. The level of parents’ education regarding dental care in T1D group was medium and low, whereas the control group dominated with the medium and higher levels of parents’ education.

### Salivary Microbiome

#### Sequencing Data

A total of 762,156 reads were obtained from sequencing with an average of ∼11.5 thousand reads per sample (ranging from 5,876 to 42,528 reads). Sequencing data passed quality check using FastQC^7^ with default parameters. Following BURST taxonomic alignments 690,143 raw reads were mapped with an average of 10,456 reads per sample (ranging from 5,482 to 26,107 reads per sample). After removing bacteria with less than 25 reads across all samples at the genus level, 689,850 reads remained for further analysis with an average of 10,452 reads per sample (ranging from 5,478 to 26,106 reads). After similar filtering at the species level, 689,399 reads remained for further analysis with an average of 10,445 reads per sample (ranging between 5,474 and 26,101 reads per sample).

#### Microbiome Characterization

**FIGURE 1:**
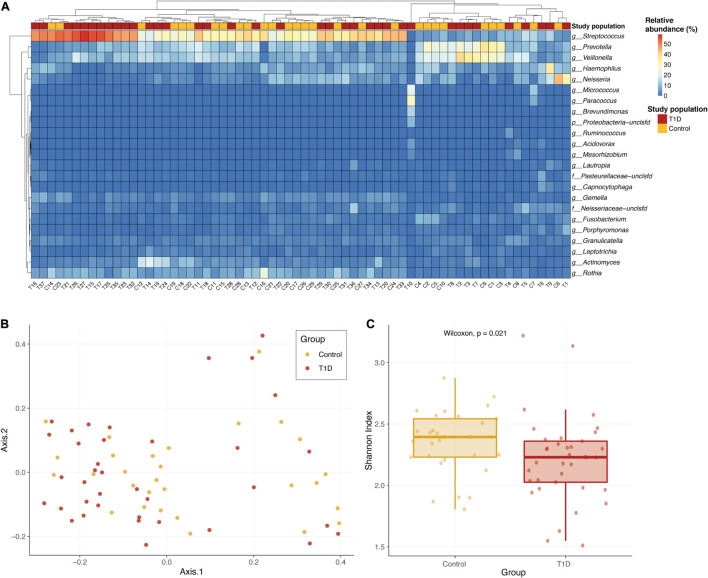
Overall microbial composition in the two population groups. **(A)** Relative abundance of the most abundant genera in the saliva of T1D group (red) and the control group (yellow). The color of each cell in the heatmap is related to the abundance level of each genera per sample. **(B)** PCoA of the Bray–Curtis dissimilarity between study samples. Samples from T1D and controls are colored in red and yellow, respectively. Samples that are closer in their microbiome composition will be closer in this 2D plot. No clear distinction between the groups is identified. **(C)** Alpha diversity comparison of microbial communities of saliva samples from T1D group (red) and control group (yellow). Shannon diversity index was calculated as the metric for alpha diversity. The boxes represent the interquartile range (IQR) between the first and third quartiles (25th and 75th percentiles, respectively) and the vertical line inside the box defines the median. *P*-value calculated using Wilcoxon test.

We found 105 different genera and 211 different species in the oral microbiome of the tested children. Most abundant genera in the saliva of both groups were *Streptococcus*, *Prevotella*, *Veillonella*, *Haemophilus*, and *Neisseria*. We first wanted to check whether the bacterial communities of the two study populations were similar or not. Overall, there are no strong shifts between the two sample types, and the most abundant genera in the saliva of both groups are *Streptococcus*, *Prevotella*, *Veillonella*, *Haemophilus*, and *Neisseria* (Figure 1A). Performing a principal coordinate analysis (PCoA) on these samples did not reveal any clear separation between the groups (Figure 1B). However, when we examined the microbial richness of each sample, we found that control samples had a significantly higher diversity (calculated using Shannon diversity index, Figure 1C).

**FIGURE 2:**
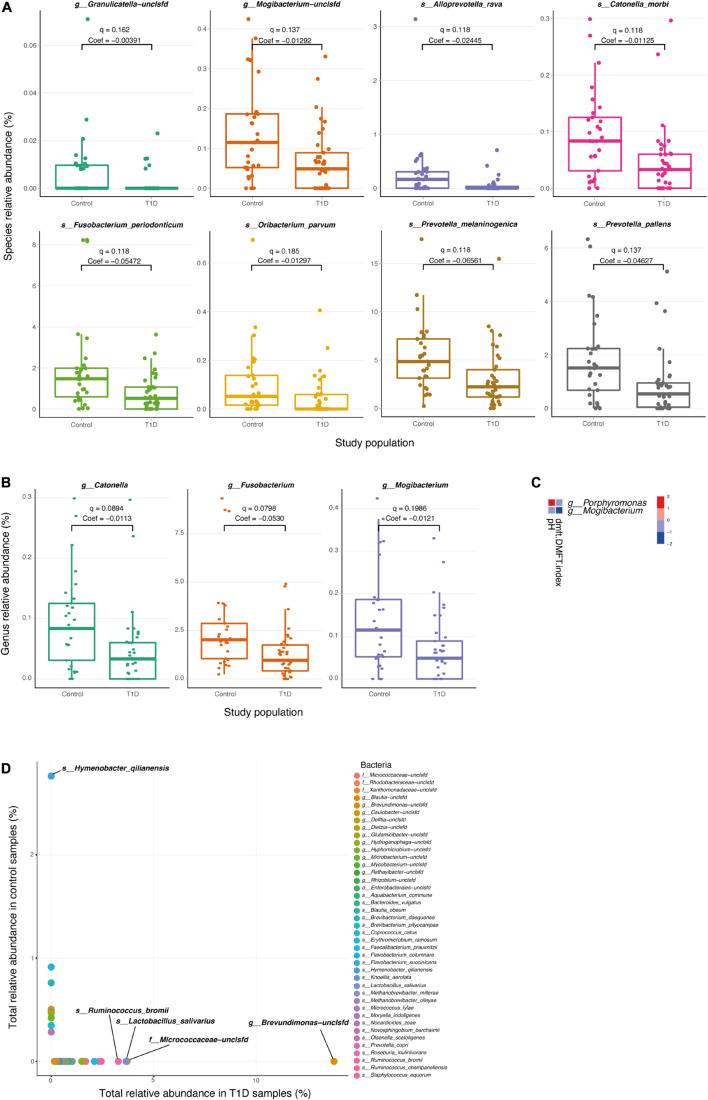
Differential microbial taxa between T1D samples and controls. **(A,B)** Relative abundance of the significant taxa at the species **(A)** and genus **(B)** level, in T1D samples and control samples. *Q*-values and coefficients calculated using a multivariate linear regression model (MaAsLin, see section “Materials and Methods). The boxes represent the interquartile range (IQR) between the first and third quartiles (25th and 75th percentiles, respectively) and the vertical line inside the box defines the median. **(C)** Significant associations between microbial taxa and clinical parameters of the oral cavity. Only salivary pH and DMFT index are shown in the vertical axis as significantly correlated parameters (*q* < 0.25), and two genera; *Porphyromonas* and *Mogibacterium*, are shown in the horizontal axis. The variations in color are the magnitude of correlation between both variables. Correlation coefficient values range between −1.0 and 1.0; a correlation coefficient that is greater than zero indicates a positive relationship between two variables, a value that is less than zero signifies a negative relationship between two variables. **(D)** Relative abundance species apparent in only one type of population. The horizontal axis represents the sum of the relative abundance of the bacteria in T1D samples. The vertical axis represents the sum of the relative abundance of the bacteria in control samples.

We next searched for differential taxa between the T1D and control samples. Using a multivariate linear regression model, we identified eight differential species and three differential genera (see section “Materials and Methods). Eight species had significantly higher values among healthy children than in T1D children (Table 1 and Figure 2A), and at the subspecies level, eight taxa were higher in the control group than T1D group (Table 1). Three bacterial genera were higher in the control group than in T1D (Figure 2B) including *Catonella* (*p* = 0.0017, *q* = 0.0894), *Fusobacterium* (*p* = 0.0007, *q* = 0.0798), and *Mogibacterium* (*p* = 0.0056, *q* = 0.1986).

**TABLE 1:** Eight species and eight subspecies had significantly higher values among healthy children than in T1D children.

*Species*	*p*	*q*
*Granulicatella-unclassified*	0.00537	0.162
*Mogibacterium-unclassified*	0.00391	0.137
*Alloprevotella rava*	0.00154	0.118
*Catonella morbi*	0.00171	0.118
*Fusobacterium periodonticum*	0.00101	0.118
*Oribacterium parvum*	0.00701	0.185
*Prevotella melaninogenica*	0.00223	0.118
*Prevotella pallens*	0.00374	0.137

***Subspecies***	***p***	***q***

*Granulicatella-unclassified*	0.00537	0.165
*Mogibacterium-unclassified*	0.00390	0.140
*Alloprevotella rava-unclassified*	0.00154	0.120
*Fusobacterium periodonticum-unclassified*	0.00101	0.120
*Catonella morbi atcc 51271*	0.00171	0.120
*Oribacterium parvum acb1*	0.00701	0.188
*Prevotella melaninogenica atcc 25845*	0.00223	0.120
*Prevotella pallens atcc 700821*	0.00374	0.140

To clarify the relationship between the changes in the salivary microbiome and other salivary parameters, we analyzed the correlations between metadata and different microbes. We found that some physiological parameters (salivary pH and DMFT index) were associated with two genera of microbes (*Porphyromonas* and *Mogibacterium*; Figure 2C).

Finally, the diabetes group presented 22 taxa at the genus level and 33 taxa at the species level that were not presented in the control group, and the control group exhibited six taxa at the genus level and nine taxa at the species level that were not present in the diabetes group (Figure 2D and Supplementary Table 1).

The five most abundant genera in the T1D group were *Brevundimonas*, *Ruminococcus*, *Micrococcaceae-unclassified*, *Blautia*, and *Faecalibacterium* with sum of values 13.79, 5.60, 3.70, 2.57, and 2.0, respectively. The most abundant species in the T1D group were *Brevundimonas-unclassified*, *Micrococcaceae-unclassified*, *Lactobacillus salivarius*, *Ruminococcus bromii*, *Prevotella copri*, *Ruminococcus champanellensis*, *Faecalibacterium prausnitzii* with sum of values 13.79, 3.70, 3.65, 3.27, 2.43, 2.33, and 2.10, respectively. The most abundant genera in the control group were *Hymenobacter*, *Xanthomonadaceae-unclassified*, *Dietzia*, *Microbacterium*, and *Erythromicrobium* with sum of values 2.76, 0.50, 0.46, 0.43, and 0.34. The most abundant species in the control group were *Hymenobacter qilianensis*, *Flavobacterium columnare*, *Brevibacterium daeguense*, *Xanthomonadaceae-unclassified*, *Methanobrevibacter olleyae*, *Bacteroides vulgatus*, and *Dietzia-unclassified* with sum of values 2.76, 0.91, 0.76, 0.50, 0.47, 0.46, and 0.42.

### Physiological Measures

#### Salivary Flow Rate

The average (±SE) of diabetic and healthy children were 0.50 ± 0.04 ml/min and 0.53 ± 0.03 ml/min, respectively, with no difference between the groups (*p* = 0.47) (Table 2).

**TABLE 2:** Salivary parameters in children with type 1 diabetes mellitus and healthy children.

Parameters	Diabetics		Non-diabetics		*P*-value
	N	Average ± SE	N	Average ± SE	
Salivary flow rate (ml/min)	37	0.50 ± 0.04	29	0.53 ± 0.03	0.47
DMFT index	37	6.08 ± 0.61	29	3.76 ± 0.67	0.01*
pH	37	6.88 ± 0.11	29	7.14 ± 0.10	0.24
Calcium (nmol/ml)	37	1.37 ± 0.11	29	1.12 ± 0.08	0.10
Phosphate (nmol/ml)	37	4.72 ± 0.25	29	4.71 ± 0.22	0.98
Urea (nmol/ml)	37	4.30 ± 0.17	29	4.23 ± 0.18	0.77

*Significant differences.

*Salivary pH*. Salivary pH showed no difference between the two groups (*p* > 0.05), with an average (±SE) 6.88 ± 0.11 and 7.14 ± 0.10 of the experimental and control group, respectively.

#### Salivary Glucose

The percentage of salivary glucose concentrations showed 95 and 100% negative results in diabetic and healthy children, respectively (Table 3).

**TABLE 3:** Salivary glucose percentage in children with type 1 diabetes mellitus and healthy children.

Parameters	Diabetics		Non-diabetics	
		Percentage		Percentage
Glucose	Negative*	95	Negative	100
	Normal*	5	Normal	0

*Using Medi-Test Combi 3A where the color fields correspond to the following ranges of glucose concentrations: neg. (yellow), neg. or normal (greenish), 2.8, 8.3, 27.8 ≥ 55.5 mmol/L.

#### Salivary Calcium, Phosphate, and Urea

There were no differences in the average (±SE) values of salivary calcium (1.37 ± 0.11 and 1.12 ± 0.08 nmol/μl, *p* = 0.10), phosphate (4.72 ± 0.25 and 4.71 ± 0.22 nmol/μl, *p* = 0.98), and urea (4.30 ± 0.17 and 4.23 ± 0.18 nmol/μl, *p* = 0.77) in diabetic and healthy children, respectively.

#### Decayed, Missing, and Filled Teeth Index

Clinical examination showed higher caries incidence in diabetic children. The average (±SE) values with respect to DMFT index were 6.08 ± 0.61 and 3.76 ± 0.67 in the experimental and control group, respectively (*p* < 0.05). Furthermore, diabetic females had more tooth decay with no statistically significant difference between the groups (DMFT = 6.45 compared with 5.65 among diabetic males, *p* = 0.52). As reported from the data gathered while interviewing patients and parents, diabetic patients had poor quality diet, poor oral hygiene, less access to dental care, and less parents’ dental education compared with the control group.

## Discussion

Only a limited number of studies have investigated the oral microbial composition of patients with T1D. We examined the oral microbiome in children with T1D and healthy children and found significant differences between the oral microbiota of diabetic children and the oral microbiota of healthy children. This is in accordance with de Groot et al. (2017) who found a markedly difference in oral microbiota in T1D (e.g., abundance of Streptococci) compared with healthy controls.

A recently published study by Pachoński et al. (2021) using classical methods routinely used in microbiological diagnostics confirmed quantitative and qualitative significant difference between the oral microbiome of children with T1D and healthy children. The present study used salivary samples, which according to Pachoński et al. (2021) are much more diverse than the samples they acquired with swab technique in the soft tissue of the oral cavity.

As in Pachoński et al. (2021)’s study, *Streptococcus* genus was also one of the largest groups of isolated microorganisms in the present study. However, we additionally found a large amount of *Prevotella*, *Veillonella*, *Haemophilus*, and *Neisseria*. Significantly higher number of bacteria from the *Streptococcus* genus were found in the group of children with well-controlled diabetes mellitus compared with healthy children in Pachoński et al. (2021) and in the present study. Our study shows that 16S rRNA gene-based analysis enables the identification of a very broad scope of organisms: 105 different genera and 211 different species. The present study was also able to establish a unique group of bacteria (taxon) at the genus level and at the species level that either appeared or were absent in the saliva of T1D children. In addition to species and subspecies, clinical microbiologists study bacterial genera and families, so we concentrated on the differences between healthy and diabetic children in those taxonomic groups. The present data exhibited significant increase of the genera *Catonella*, *Fusobacterium*, and *Mogibacterium* in the control group. At the species level, *Granulicatella* spp., *Alloprevotella rava*, *Catonella morbi*, *Fusobacterium periodonticum*, *Oribacterium parvum*, *Prevotella melaninogenica*, and *Prevotella pallens* were significantly more abundant in the control group, in addition to *Catonella morbi ATCC 51271*, *Oribacterium parvum ACB1*, and *Prevotella melaninogenica ATCC 25845* at the subspecies level. *Brevundimonas*, *Ruminococcus*, *Micrococcaceae* spp., *Blautia*, and *Faecalibacterium* were predominant genera only in the T1D group.

According to the literature, three of these subspecies—*Blautia*, *Ruminococcus* (of family Lachnospiraceae), and *Faecalibacterium*—were enriched in women with gestational diabetes (Crusell et al., 2018).

As reported in the Results section, several unique taxa were identified in T1D: *Lactobacillus salivarius*, *Ruminococcus bromii*, *Prevotella copri*, *Ruminococcus champanellensis*, and *Faecalibacterium prausnitzii.* The identification of those unique taxa can be partly clarified by the quality of diet consumed by diabetic patients, in addition to the increase of periodontal inflammation among T1D children (Novotna et al., 2015). Diet has an important role in composition and metabolism of the oral microbiome. We report poor-quality diet among T1D children, which is in line with previous reports (Patton, 2011) of high saturated fat consumption and low intake of fruits, vegetables, and whole grain foods. Such diets are rich in advanced glycation end products (AGEs), which are complex heterogeneous compounds derived from non-enzymatic glycation reactions. While dietary advanced glycation end products (dAGEs) are formed during industrial processing and home cooking, high plasma glucose, as in diabetes (Rhee and Kim, 2018), accelerates the formation of endogenous AGEs. AGE-modified proteins accumulate within the body and are thought to play a role in a number of age-related diseases including diabetes. Long-term glycemic control regime decreased AGE levels in patients with T1D (Kostolanská et al., 2009).

As the absorption of dietary AGEs is limited, the majority of protein-bound AGEs pass through the gastrointestinal tract to the colon, where they can serve as substrates for the gut microbiota. Conflicting evidence on how dietary AGEs influence the composition of the microbiome include reduced levels of *Prevotella copri*, found here to be predominant genera only in the T1D group in peritoneal dialysis patients who underwent a 4-week low-dAGE regime (Yacoub et al., 2017) and that an AGE-rich diet in rats reduced the abundance of *Ruminococcaceae* and *Alloprevotella*, genera that we found to be both predominant and exclusive to the T1D group. The same diet increased the levels of *Bacteroides* (found in our study only in the control group) (Qu et al., 2017).

Elaboration on the unique taxa found in T1d patients in our study:

(1) *Brevundimonas* spp. are non-fermenting Gram-negative bacteria considered of minor clinical importance infection. Many of these non-fermenting Gram-negative bacteria are opportunistic pathogens that affect patients suffering from underlying medical conditions (Ryan and Pembroke, 2018) including diabetes (Lee et al., 2011). In the oral cavity, *Brevundimonas diminuta* was detected in refractory periodontitis (Krishnan et al., 2017).
(2) *Blautia* and *Faecalibacterium prausnitzii* are members of the human gut microbiome producing butyrate as fermentation end product. A high concentration of butyrate could result in apoptosis in human gingival epithelial cells and play an essential role in the initiation of periodontitis (Guan et al., 2021). Abundances of *Faecalibacterium* were negatively correlated with HbA1c levels in T1D (Huang et al., 2018). Moreover, a relative overabundance of the genus *Blautia* was found in the gut microbiome in the prediabetes and progressive stage of T1D (Kostic et al., 2015).
(3) *Prevotella copri* is by far the most abundant member of the genus *Prevotella* inhabiting the human large intestines. *P. copri* is strictly dependent on a sugar source partly elucidating its detection in T1D individuals in our cohort, who reported poor quality and high sugar diet. In the oral cavity, the proportion of *P. copri* was relatively higher in T2D patients with periodontitis (Sun et al., 2020). Thus, the detection of *Brevundimonas*, *Blautia*, *Faecalibacterium prausnitzii*, and *P. copri* in the oral cavity of T1D children may be associated with altered periodontal state among the study group.
(4) *Ruminococcus* is a genus of gut microbiome. Experimental evidence has confirmed its significant difference in the gut of diabetic mice may contribute to the pathogenesis of T1D by decreasing FOXP3-positive regulatory T cells (Tregs) that protect against diabetes (Krych et al., 2015). *R. bromii* possesses an exceptional ability to colonize and degrade starch particles in the human colon (Crost et al., 2018). *R. champanellensis* is a cellulose-degrading bacterium from human gut microbiota, in which fermentable carbohydrates are required for growth of this species (Chassard et al., 2012). Thus, the presence of *Ruminococcus* species in the saliva of T1D children in our cohort might be due to high intake of starch and sugars fermented by these bacteria.
(5) *Lactobacillus* is an indigenous member of human gut and oral microbiota. *L. salivarius* was found to be more highly associated with caries in children than the other *lactobacilli* because it is acidogenic and can produce lactate, acetate, and hydrogen peroxide (Piwat et al., 2010). Thus, we suggest that the elevated caries incidence among T1D children could induce increase of *L. salivarius* in the study group.

When checking the taxa found in the present study against data on T1D in gutMDisorder,^8^ a manually curated database of comprehensive dysbiosis of the gut microbiota (Cheng et al., 2020), the genus *Blautia* increased in the gut microbiome of T1D patient and was present only in the T1D group in our study. Genus *Haemophilus* and family *Veillonellaceae* were abundant in both groups in the present study compared with a decrease in the gut microbiome of T1D patients. *Fusobacteria* phyla were more abundant in the control group of the present study and were decreased in gut microbiome. *Porphyromonadacea* species were increased in the gut of T1D patients and appeared to be correlated with oral parameters in the present study. Genus *Prevotella* was abundant in both groups in the present study compared with a decrease in the gut microbiome of T1D patients. Genus *Bacteroides* was increased in T1D gut microbiome, but *Bacteroides vulgatus* was found only in the saliva of the control group in the present study. Being part of the typical westernized pattern, *Bacteroides*, *Faecalibacterium*, and *Prevotella* were the predominant genera in gut microbiota composition of both women with gestational diabetes and normal glucose regulation (Crusell et al., 2018). Although we find similarities between gut and oral microbiome, there appear to be multifaceted relations that are determined by the environment; genera *Porphyromonas* and *Mogibacterium* were correlated with both pH and DMFT index parameters and were both classified as microbial signatures of periodontitis in the oral microbiome (How et al., 2016; Hunter et al., 2016). There are studies that show a lower incidence of dental caries in diabetic children compared with their healthy peers (Orbak et al., 2008), differing from our study and from others (Ferizi et al., 2018) who presented a significantly higher DMFT index in children with T1D than that in the control. This can be related to the fact that in both studies children with T1D rarely visited the dentist. In addition, in the present study children in the control group were orthodontic patients who kept high standards of oral care.

No differences in unstimulated salivary flow rate and salivary glucose calcium and phosphate levels were detected between the two groups. Furthermore, no significant difference was observed between diabetic and healthy children with respect to salivary urea, in disagreement with López et al. (2003) who found greater salivary urea levels in T1D children than in controls.

### Study Limitation

Like Pachoński et al. (2021), the present study was a preliminary one and was not aimed to link between quality of dental care and oral hygiene and differentiating the dental health status between the children with T1D and healthy controls. Differentiating the oral microbiome in this case will be targeted by a more specific future study. Because this was an initial study examining the differences in oral microbiome between children with type 1 diabetes and non-diabetes children no power calculation was performed. It was decided to collect saliva from all attendants to the division of Pediatric Endocrinology, Hadassah Medical Center, Hebrew University of Jerusalem, Israel, who met the inclusion criteria and were willing to participate in the study during a period of 1 year.

## Conclusion

We have established a unique microbial taxon that either appeared or were absent in the saliva of T1D children.

Many of the bacteria identified belong to the gut microbiome, indicating the complex interplay between the oral and gut microbiome in the pathogenesis of T1D.

In addition, some microbial taxa were linked to other parameters in the oral cavity of T1D individuals, such as higher incidence of dental caries.

## Data Availability Statement

The datasets presented in this study can be found in online repositories. The names of the repository/repositories and accession number(s) can be found below: NCBI SRA BioProject, accession no: PRJNA759836.

## Ethics Statement

The studies involving human participants were reviewed and approved by the Institutional Human Subjects Ethics Committee of Hadassah Medical Organization (0714-18-HMO). Written informed consent to participate in this study was provided by the participants’ legal guardian/next of kin.

## Author Contributions

MM, SF, DZ, DR, and DS conceived and designed the study. MN collected the samples. MN and AC performed laboratory assays. MY and NM performed bioinformatics analysis. MN and MM performed statistical analysis and wrote the draft of the manuscript. MM, DS, and MN interpreted the results. MM and DS supervised the work and revised and contributed to the final manuscript. DR contributed with resources and funding. All authors read and approved the final article.

## Conflict of Interest

The authors declare that the research was conducted in the absence of any commercial or financial relationships that could be construed as a potential conflict of interest.

## Publisher’s Note

All claims expressed in this article are solely those of the authors and do not necessarily represent those of their affiliated organizations, or those of the publisher, the editors and the reviewers. Any product that may be evaluated in this article, or claim that may be made by its manufacturer, is not guaranteed or endorsed by the publisher.

## Footnotes

1
https://github.com/knights-lab/BURST/releases/download/v0.99.7f/burst_linux_DB12

2
https://www.ncbi.nlm.nih.gov/refseq/targetedloci/

3
https://CRAN.R-project.org/package=vegan

4
https://CRAN.R-project.org/package=ggpubr

5
https://github.com/knights-lab/BURST

6
https://www.bioconductor.org/packages/release/bioc/html/Maaslin2.html

7
https://www.bioinformatics.babraham.ac.uk/projects/fastqc/

8
http://bio-annotation.cn/gutMDisorder/browse.dhtml
