# Supplementary material for: Characterization of the Oral Microbiome Among Children With Type 1 Diabetes Compared With Healthy Children
Source: Front Microbiol. 2021 Oct 29;12:756808. doi: 10.3389/fmicb.2021.756808 (PMC8586508; doi:10.3389/fmicb.2021.756808)
Supplement: Supplementary file 1 [file Data_Sheet_1.docx]

**Supplementary material**

**Table S1.** Taxa apparent in only one population at the genus level **(a)** and species level **(b)**. The “Sum of Values” column refers to the sum of the relative abundance of the bacteria within a given sample in the control and T1D groups. The number of samples included for each type of bacteria had a value greater than 0%, and either the control sum or the T1D sum must be 0 to follow the criteria of inclusion. The files are ordered such that the bacteria with a bigger sum of values (either in T1D samples or in control samples) are at the top of the table.

| **Bacteria** | **Control_Sum_of_ Values** | **T1D_Sum_of_ Values** | **Number_of_Samples_Over_ Zero** |
| --- | --- | --- | --- |
| **g__Brevundimonas** | 0 | 13.80 | 1 |
| **g__Ruminococcus** | 0 | 5.61 | 1 |
| **f__Micrococcaceae-unclsfd** | 0 | 3.70 | 1 |
| **g__Hymenobacter** | 2.76 | 0 | 1 |
| **g__Blautia** | 0 | 2.58 | 2 |
| **g__Faecalibacterium** | 0 | 2.11 | 1 |
| **g__Roseburia** | 0 | 1.68 | 2 |
| **g__Hydrogenophaga** | 0 | 1.49 | 2 |
| **g__Methanobrevibacter** | 0 | 0.93 | 1 |
| **f__Rhodobacteraceae-unclsfd** | 0 | 0.81 | 3 |
| **g__Aquabacterium** | 0 | 0.80 | 2 |
| **g__Rathayibacter** | 0 | 0.75 | 1 |
| **g__Knoellia** | 0 | 0.74 | 1 |
| **g__Coprococcus** | 0 | 0.70 | 1 |
| **g__Glutamicibacter** | 0 | 0.68 | 1 |
| **g__Hyphomicrobium** | 0 | 0.64 | 1 |
| **g__Mycobacterium** | 0 | 0.60 | 1 |
| **g__Delftia** | 0 | 0.58 | 2 |
| **o__Enterobacterales-unclsfd** | 0 | 0.57 | 2 |
| **g__Novosphingobium** | 0 | 0.55 | 1 |
| **f__Xanthomonadaceae-unclsfd** | 0.50 | 0 | 1 |
| **g__Dietzia** | 0.47 | 0 | 1 |
| **g__Microbacterium** | 0.430 | 0 | 1 |
| **g__Rhizobium** | 0 | 0.39 | 1 |
| **g__Erythromicrobium** | 0.35 | 0 | 1 |
| **g__Nocardioides** | 0.286 | 0 | 1 |
| **g__Moryella** | 0 | 0.19 | 1 |
| **g__Caulobacter** | 0 | 0.16 | 2 |

| **Bacteria** | **Control_Sum_ of_Values** | **T1D_Sum_of_**  **Values** | **Number_of_Samples**  **_Over_Zero** |
| --- | --- | --- | --- |
| **g__Brevundimonas-unclsfd** | 0 | 13.79 | 1 |
| **f__Micrococcaceae-unclsfd** | 0 | 3.70 | 1 |
| **s__lactobacillus_salivarius** | 0 | 3.65 | 6 |
| **s__ruminococcus_bromii** | 0 | 3.27 | 1 |
| **s__hymenobacter_qilianensis** | 2.76 | 0 | 1 |
| **s__prevotella_copri** | 0 | 2.43 | 2 |
| **s__ruminococcus_champanellensis** | 0 | 2.33 | 1 |
| **s__faecalibacterium_prausnitzii** | 0 | 2.11 | 1 |
| **s__roseburia_inulinivorans** | 0 | 1.68 | 2 |
| **g__Blautia-unclsfd** | 0 | 1.55 | 2 |
| **s__Brevibacterium_pityocampae** | 0 | 1.49 | 1 |
| **g__Hydrogenophaga-unclsfd** | 0 | 1.49 | 2 |
| **s__blautia_obeum** | 0 | 1.02 | 1 |
| **s__staphylococcus_equorum** | 0 | 0.93 | 1 |
| **s__flavobacterium_columnare** | 0.91 | 0 | 1 |
| **s__flavobacterium_succinicans** | 0 | 0.87 | 1 |
| **f__Rhodobacteraceae-unclsfd** | 0 | 0.81 | 3 |
| **s__aquabacterium_commune** | 0 | 0.80 | 2 |
| **s__brevibacterium_daeguense** | 0.76 | 0 | 1 |
| **g__Rathayibacter-unclsfd** | 0 | 0.75 | 1 |
| **s__knoellia_aerolata** | 0 | 0.74 | 1 |
| **s__coprococcus_catus** | 0 | 0.70 | 1 |
| **g__Glutamicibacter-unclsfd** | 0 | 0.68 | 1 |
| **g__Hyphomicrobium-unclsfd** | 0 | 0.64 | 1 |
| **g__Mycobacterium-unclsfd** | 0 | 0.60 | 1 |
| **g__Delftia-unclsfd** | 0 | 0.58 | 2 |
| **o__Enterobacterales-unclsfd** | 0 | 0.57 | 2 |
| **s__novosphingobium_barchaimii** | 0 | 0.55 | 1 |
| **f__Xanthomonadaceae-unclsfd** | 0.50 | 0 | 1 |
| **s__methanobrevibacter_olleyae** | 0 | 0.50 | 1 |
| **s__bacteroides_vulgatus** | 0.48 | 0 | 3 |
| **g__Dietzia-unclsfd** | 0.47 | 0 | 1 |
| **s__methanobrevibacter_millerae** | 0 | 0.43 | 1 |
| **g__Microbacterium-unclsfd** | 0.42 | 0 | 1 |
| **g__Rhizobium-unclsfd** | 0 | 0.39 | 1 |
| **s__erythromicrobium_ramosum** | 0.35 | 0 | 1 |
| **s__nocardioides_zeae** | 0.28 | 0 | 1 |
| **s__micrococcus_lylae** | 0 | 0.27 | 1 |
| **s__olsenella_scatoligenes** | 0 | 0.24 | 1 |
| **s__moryella_indoligenes** | 0 | 0.19 | 1 |
| **g__Caulobacter-unclsfd** | 0 | 0.16 | 2 |
